# Wheat Phosphates

**Published:** 1882-01

**Authors:** William S. Daffin


### WHEAT PHOSPHATES.

BY WILLIAM S. DAFFIN, M. D.

No one is more ready than the writer to admit that the compounds of phosphorous have been run into a very quackish channel—that the methods of certain manufacturing chemists have done decidedly more injury than good to a very valuable class of remedial agents. But their unprofessional, if not dishonorable, conduct has not deteriorated the value of the remedies. Wheat phosphates sold under a woman's name are just as efficient as they would be if they had the name of the New York professor attached to them—he who so fortunately pockets a fair income from their sale. I see in some of the professor's advertisements (for they appear alike in hand bills, railroad guides, police gazettes and other secular journals, as well as in first class medical journals) that he claims that his nostrum will stamp out consumption, renovate old men, bless the arms of disconsolate Sarahs with joyous babes, and almost entirely rob the King of Terrors of his victim. Such quackery, such falsehoods and ridiculous assertions, even if coming from a distinguished professor and prize essayist, tends to disgust the profession with this class of remedies. Notwithstanding all this, wheat phosphates do possess valuable properties which are not sufficiently recognized by the profession. To enumerate them and to excite an interest in them I indite this paper. I do not believe they will cure consumption, but numerous observations have satisfied me that they are by no means so worthless as the ordinary laboratory preparations. Dr. Anderson, in his work, "Phosphates in Nutrition," has demonstrated the presence in the capillaries of a peculiar blending of the phosphates of lime, magnesia and potash, which he terms the tissue phosphates. The tissue phosphate he has tested as an anti-phthisical remedy, and in the book above referred to, reports some very gratifying results with it. An almost identical phosphate is found in the inner shell of wheat. If isolated so as to form the identical combination in which it normally exists, it would be the par excellent remedy for phosphatic deficiency. Unfortunately, by the crude methods usually pursued the normal combination is destroyed, and the individual phosphates are "but single stones detached from the great mosaic picture," as the world-known Hudicum has so aptly said. But only one or two individuals have, so far, succeeded in procuring the normal phosphates. Perhaps Anderson and Polk are the only ones, and they both have maintained an unprofessional reticence on the methods of isolation. The so-called "vitalized phosphates" is said to be only a by product in the manufacture of "Hecker's Self-Raising Flour," which costs the "Professor" about six cents a pound. Selling for eight dollars leaves a handsome profit for himself and the lady whose name appears on the label as "instructed most minutely in the preparation of these delicate chemicals," the knowledge required being to fill, cork, label and wrap the bottle, the Hecker Company doing the rest of the work. Fishblatt, in the February, 1880, number of his *New York Medical and Surgical Journal*, so completely exposed this fraud that I need not consider it further.

It may seem useless to enlarge upon the organismal value of the phosphates. Their ubiquity and essentiality in both the animal and vegetable kingdoms, the high offices they sustain in both these kingdoms, are now recognized by the chemist, the physician and the agriculturist. No stronger testimony of their influence upon the growth and development of vegetable life is needed than the rapidly increasing use of the phosphates by agriculturists. The manufacture of phosphates for this purpose has become one of the most important industries of the country. By them, wornout land has been renovated and made productive; already good land has been stimulated to double its yield. Chemical analysis explains why this occurs; the phosphates are abundant in all vegetable structures, be it grass, vegetables, cereals, or timber. The phosphates abound in every thing that lives, and justify the aphorism of Horsford, "without phosphoric acid, there is no life." If the aphorism be true that phosphoric acid is inseparable from animal and vegetable life, a deficiency in amount must be attended with a corresponding depreciation of vital force. We well know that a deterioration of vital force is always in itself a strong predisposition to disease; that morbific influences, which would be overcome by a person in the enjoyment of health, develop disease in the debilitated. But there can not be any doubt but that phosphatic deficiency has a more intimate relation to disease than a mere predisposing cause. Human bone and teeth are mainly composed of phosphate of calcium and magnesium. From these they acquire their durability and strength. If the amount falls below the normal standard, the disease known as rickets is developed. The remedy is the restoration of the deficient phosphates. In both tuburculous and scrofulous diseases there is an exaggerated waste of the phosphates, the quantity excreted often rising fifty per cent. above the normal quantity found in the urine. This loss must be a heavy strain upon the integrity of the organism. Can we not connect it with one of the most formidable lesions of these maladies, the hectic fever which so fearfully burns up tissues and hastens the progress of disintegration. I do not claim that all hectic fever arises from this source. Every thinking physician is aware that much of it is the direct result of pus poisoning, but its frequent occurance when there is no tyromatous tubercles, or inflammatory exudations, and yet an inordinate phosphatic waste points to the latter as the probable cause. Clinical results also sustain this hypothesis. Whatever checks the phosphatic waste, reduces the fever. This frequently follows the administration of anti-phthisical remedies, as cod-liver oil, crude petroleum, extract of malt, and the hypophosphites, especially the organismal kind. But this evidence is not quite so conclusive as that which follows the administration of the wheat and animal phosphates. While they do not influence as much as do others the progress of the disease, they diminish the fever more promptly than any other remedy I have ever used. The argument would be plausible in regard to the others, that the remedies, by counteracting the course of the disease, thus lessened the fever, but in the case of the phosphates it is different. They act as a powerful anti-febrile remedy, reducing the pulse, lessening the number of respirations, producing a lower temperature, and increasing the strength without dininishing the amount of the phosphates in the urine, showing the direct connection between these symptoms and phosphatic deficiency. In a short time, however, the phosphatic secretion is diminished, demonstrating that the organism or wheat phosphates do exercise a potent power in the control of this malady. On the other hand, if the ordinary phosphates be used no such result occurs. This difference in the value of the two classes has been recognized by many eminent physicians.

Dr. Tilbury Fox, a leading physician of London, and well known as an author on "Skin Diseases," says: "There is something special in the organized phosphates, those in fact which have been formed by passing through a living organism as compared with those artificially prepared."

"It is not the *amount*, but the *kind* exhibited which produces the good result. In infant's food, and in our bread and flour, the organized phosphates and cerealine (which has somewhat similar action to pepsin) have been deliberately rejected. These may be administered medicinally to children and infants when the assimilative function is at fault. In eruptive diseases of the scalp (which are generally associated with faulty assimilation), Rickets, Morasmus, Chronic Diarrhœa, and impaired nutrition of all kinds, the wheat phosphates act marvellously. Pallid children pick up tone, color, and flesh, worms disappear, the secretions become healthy, and diseases go."

M. Andre Sanson, in a paper read at the Academy of Medicine (Gazette Medicale, August 14th, 1860) says: "That to promote osseous growth, the administration of phosphate of lime, either in the shape of hypophosphites (laboratory kind), or in that of powdered bone is unavailing. Several attempts made with these substances have never been successful. *It is because their form does not allow of their assimilation. On the contrary, organic phosphates, such as are elaborated in vegetables, are real aliments.*"

Dr. Bouchardat says: "There is the difference between all laboratory compounds and organic compounds that is found to exist between natural mineral water and a fabricated water."

Dr. Begin, in his "Essay on Wine," page 11, says: "Nature possesses means of preparation and secrets of the alembic which it is impossible for science to attain, and which furnish the healing art with agents much more efficacious than those prepared by chemistry. It is thus with iron, which in the different forms in which it is tried is not assimilable; it either escapes digestive action or it produces such irritating and exciting effects in the organism that its use can not be long employed. The same with iodine and other bodies, the action of which may be great and their direct use produce many inconveniences, and often danger.

"If, on the contrary, you appeal to Nature and not to chemistry, iron and iodine are met with in perfectly assimilable forms, and in such condition that they agree with the economy of our nature without fear of accident, repulsion or elimination."

Sir Henry Thompson said in the London *Lancet*, January 13th, 1873: "Thus, for example, you know that you may give A an ounce of salts, or B half an ounce, and you purge them; but you may obtain the same effect with one-fifth of those quantities, if you give it as prepared in Nature's laboratory, in the form of mineral water. It is a curious fact, which I give as an ultimate one, and without speculating here on the cause of this difference. As a proof of the superior force of the saline combinations found in natural springs, I may refer you to the following experiment. If you will reduce by careful evaporation, as I have done, such mineral waters to their pharmaceutical condition of crystalized salts, you will find them possessing little, if any more power than similar salts as obtained by the ordinary processes, and met with in every chemist's shop. They no longer do their work on the same terms as when administered in the original water, before they were separated by evaporation. You will, therefore, readily understand how essential to our end it is to employ the *natural* mineral waters, since what are called "*artificial waters*," however admirably prepared, are simply pharmaceutical products, and *are destitute of the very quality* which distinguishes the remedies they are designed to imitate."

Dr. Bryce, editor of the *Southern Clinic*, thus spoke in an editorial of the Organismal Phospheide "Glycerite of Kephaline:" "It will be found to be a powerful nutrient tonic, and a remedy of great value in tubercular phthisis, loss of memory, decay of the brain powers, neuralgia, loss of virile power, sleeplessness, general vital deterioration; the various mucous fluxes, as leucorrhœa, uterine catarrh, etc.; as also on the general debility of the female sex. We have obtained most excellent results from the use of this remedy."

Dr. Goss, in his work on "New Medicines," page 119, says: "A few months after Churchill introduced his hypophosphites, Dr. Polk finding Churchill's theory of tuberculosis to be correct, as far as the cause was concerned, but inefficient in his therapeutics, determined to test the phosphorous principles as they exist in the brain of fish. He tested these brain hypophosphites in 1859, and succeeded in curing a large per cent. of cases. While Churchill failed of a high degree of success, in consequence of using the chemically made hypophosphites, it is not so with Polk and Tilbury Fox, who used the organismal hypophosphites of the brain and of cereals; for they had the proud satisfaction to witness the most gratifying results. Under the reconstructive or morphological influence of these hypophosphites the hectic flush soon departs, the wan and emaciated form resumes its wanton vigor, cough and hemorrhages gradually lessen, and finally the patient resumes his health."

I have thus quoted from various sources and in reference to various organic compounds, and it seems to me that these quotations well establish the proposition that organic compounds are far more active and curative in their influence than the imitations formed in the laboratory; that the skill of the chemist is far exceeded by the alembic of Nature. The conclusions of indisputable authority in reference to iodine, iron, wine, the natural mineral waters and the organismal hypophosphites is equally true of wheat phosphates. They supply the phosphates in the very formula in which they exist in the capillaries and tissues. Here an objection may be urged, that the system is supplied by food with phosphates; and if it be properly prepared, it will furnish all the organism will need. To this I fully assent, if the patient be in a state of health, if the assimilative functions are in a condition capable of the necessary morphology. Unfortunately in diseased conditions the digestive powers may be so deranged as to fail to assimilate the food, and the phosphates are lost. But a very parallel case here suggests itself. In fevers the stomach often becomes incapable of digesting beef or other solid food, but at the same time has sufficient digestive powers to assimilate essence of beef and milk. So in debility or deranged conditions of the nutritive organs, the isolated wheat phosphates whether presented in the old fashion bran tea, or in a more elegant glycerated wine, may be appropriated and supply any want of the phosphates in the system, when the supply could not be obtained from the food. There is, however, another point right here presented. The value of food is largely in proportion to the amount of the phosphates which it contains, those containing a large amount of them being the most highly esteemed. Wheat, of all cereals, contains the largest amount of them, but they are not uniformly distributed through the berry, but are nearly all garnered up in the shell or bran, or in the membrane which encloses the white amylaceous portion, the gluten sacks. In preparing wheat for bread, in trying to give it a white appearance the gluten sacks, rich in nitrogenous and phosphatic constituents, are rejected. In order to improve the appearance of wheat bread, our millers have changed it from a nitrogenous to an amylaceous or carbonaceous food, with almost entire loss of the phosphates. Wheat contains about 23 parts in 1,000 of phosphates, while wheat flour contains a fraction less than one per cent. The whole potato contains about 3.32 in 1,000, while the peeled and boiled scarcely contains a trace. Boiled meats and vegetables yield up nearly all their phosphates, which are usually unconsumed. With these inordinate wastes of the phosphates, is there any wonder that phosphatic deficiency should be so prevalent, or that much vital deterioration would in consequence ensue? A writer on tuberculosis says: "Every mental operation, every physical exertion, every respiratory action of the lungs, every contraction of the heart, every digestive process of the stomach, every act of assimilation and sanguification converts phosphorus or some of its compounds into a new body; changes an atom of phosphorous into a new formula, and, in these never-ceasing variations, becomes an exalter of force and energy, imparting health, vigor and normality to every vital operation."

There are some doubting Thomases everywhere, some who reject every new truth, who view every improvement as a doubtful innovation, and even question the evidence before their own eyes. They will ask for more evidence as to the nutrient value of the phosphates, and will wish to dispute the aphorism of Horsford, "without phosphatic acid there is no life." I recall the experiment of Dr. Polk, published in his essay, "Tabes Pulmonia," 1872. I quote Dr. Polk's own language:

"In 1870 I reached the conclusion, by scientific deductions, unnecessary here to mention, that the ordinary phosphates produced in the laboratory were entirely inert as nutrients, and consequently could not be employed with success in restoring a deficiency of the phosphorous elements, or in supplying these elements in diseases which produce an excessive drain of them, as the abundant expectoration of bronchitis, catarrhal pneumonia, leucorrhœa, hæmorrhoids; abcesses, chronic ulcers, suppurating wounds, spermatorrhœa, frequently repeated sexual intercourse, and intense mental exertion, whether employed in purely intellectual labor or in the manifestations of intense emotions of love, passion, joy, and the agony of long continued grief. Having become convinced of the inefficiency of these laboratory phosphates as medicine, I determined to subject them to a practical test, to decide the accuracy or fallacy of my opinion. On the 5th of February, 1870, I placed three dogs in a kennel, gave them distilled water to drink. By water, alcohol and chloroform, I removed from their food all of its phosphatic princples, and then gave them all of this dephosphated food they could eat. For the first few days I did not perceive any change in their condition. At the termination of the first week they began to show signs that their food had been deprived of a very important constituent. While they yet ate a large amount of food, it did not seem to be digested and assimilated; even with food by them, they expressed hunger and evinced loss of flesh. Evidences of innutrition grew more obvious day by day; they grew thin, haggard—whined and told of severe suffering. On the sixteenth day they were so much emaciated that I deemed it cruel to proceed further, having demonstrated with axiomatic certainty the correctness of Professor Horsford's aphorism. "without phosphoric acid there is no life," and desisted. Upon their usual food they rapidly regained their former vigor. This experiment corroborated in its results the experiment by Professor William A. Hammond upon himself, that food deprived of its phosphatic constituents is incapable of supporting animal life. I further proved that laboratory made phosphates differ from those that enter the animal system through the natural elaboration of vegetable life, and consequently in cases of their deficiency can not compensate for them. Impressed with the importance of this discovery, I renewed my experiment on a spitzer dog. While abundantly supplied with dephosphated food, he emaciated and grew sick. I then macerated the inner or fine bran with alcohol, ether, and bisulphide of carbon, at the temperature of 120° F., precipitated the phosphates by running the temperature down to 30° F., and gave him this precipitate with his food. He at once began to improve, and in four weeks gained flesh and was every way in better health than he was when I began to subject him to this ordeal. I proved by these experiments that ordinary phosphates are entirely destitute of organismal value, consequently it is utter folly to give them to supply any deficiency or compensate for any unnatural or excessive loss of the phosphorized elements.

The results attained in these experiments seem to me to be very conclusive of the vast chasm which yawns between the organic and the inorganic world; an evidence of the peculiar impress which that mysterious essence–life stamps upon all of the processes and productions. Science may feebly imitate, but she can not evolve them. The chemistry of the laboratory is not the chemistry manifest in plants and animals. While the histologico chemist may determine the chemical forces displayed in the various vital phenomena, he can not reproduce those forces in the laboratory.

But from the experiments here given the following conclusions seem to be established beyond the possibility of contradiction:

1. The phosphorous elements hold a very important relation to nutrition. Food, however rich in its nitrogenous and carbonaceous constituents, can not sustain animal life, because the force which determines its morphology into blood, replete of tissue elements, is not exerted, consequently it can not undergo the evolution to that vital organization it must attain before it can be appropriated to the organization and reorganization of the structures of the various organs and tissues.

Hypophosphites, phosphites and phosphates manufactured in the laboratory, being destitute of organismal properties, can not compensate for the organismal phosphorous elements in the food of man and animals, and consequently should not be employed in pathological conditions in which phosphatic deficiency sustains the relation of exciting and determining cause.

Phosphates, phosphites and hyphosphites isolated from animal organisms without chemical change, being in the exact formula normal to those organisms, retain their nutrient and assimilable characters, and therefore supply those phosphorized principles in the very formula the human organism requires them, consequently they must meet absolutely and perfectly any deficiency of the phosphorous bases which may accrue from food deficient in its phosphatic constituents, or from excessive consumption or waste of these.

It is thus determined without phosphoric acid there is no life; that without it the food, however abundant in quality, is incapable of supplying the nutritive functions, and conducting those vital processes by which food is transformed into blood, and the blood is elaborated into living structures. But while the phosphates, phosphites and hyphosphites are all indispensable requisites of digestion, seeming to hold a high office in the elaboration and vitalization of the gastric juice, the part performed by the hypophosphites holds the highest relation. Developing the nerve influence exerted by the great sympatheticus, and by the medulla oblongata through the pneumogastric nerve, the whole process of nutrition is, in a large measure, subservient to its power. The evidences I have presented, the experiments I have submitted, as made by Bernard, Brown-Sequard, Hammond and myself, it seems to me are positive proof that digestion, assimilation, secretion, in fact all the organic functions of animal life are, in a large measure, preserved in their normality or thrown into abnormality, through the nerve influence communicated from the medulla oblongata. If the medulla oblongata fails to transmit the edequate force, inadequacy of function must be displayed in the organs to which it is distributed; gastric digestion will be deranged, the pancreas and liver impaired in function, butyric fermentation will ensue, and the tubercular cachexia follow in the sequence.

At the time the author wrote the above he seems to have been unaware of the previous experiments of the distinguished physiologist, Majenda. The latter fed a dog on white bread for fifty days, and starved it to death. Another dog fed on brown bread experienced no inconvenience. Both of these experimenters reach one conclusion---food deprived of its phosphates can not sustain life. Polk went a step further and proved that laboratory phosphates are innutritious, and can not replace those normal to the food, while isolated wheat phosphates, like pepsin, ptyalin and pancreative, retain organismal powers, and amply supply phosphatic deficiency. While Dr. Polk undoubtedly was the first to use isolated wheat phosphates as a medicine, the honor of introducing them into medical practice undoubtedly belongs to Dr. Tilbury Fox, the distinguished dermatologist, and really one of England's greatest medical authorities---a man almost without a peer.

From this assemblage of facts the conclusion is inescapable that the mineral constituents of food, and especially the phosphates, are necessary for the support of life and the continuance of health; that if they be deficient the vital functions become disordered and death follows.

Dr. S. B. Hunt, in his contributions to the sanitary memoirs of the late civil war, entitled Army Alimentation in relation to the Cause and Prevention of Disease says: "The various salts of lime, soda, magnesia and phosphorus are all essential to the proper assimilation of food. Starvation is a comparative term. We can starve muscles by withholding nitrogen; we can starve the fats of the bodies by withholding carbon; so we can starve the brain by withholding phosphorus, and starve the blood by failing to supply it with those salts of lime, soda, potash, magnesia and iron which are essential to its healthy condition. Just how these salts exert their power is not known, but we do know that when they are withheld the blood globule becomes irreguler in form and starvation diseases are produced."

Dr. Polk says: "The alkaline phosphates act individually in the blood, but collectively in the brain and capillaries in tissue construction. Several offices are performed by them, and these need individual examination.

"Phosphate of calcium and magnesium, with a portion of calcium carbonate, compose the bony structure, or supporting timbers of the vitalized organism. Its strength principally depends upon an adequate quantity of these earthly constituents. If, through deficient supply or excessive waste, the bones become depleted of them, osseous disease is inevitable. The limbs fail to support the weight, they become bent and deformed. * * * These phosphates also possess another important office; they increase the solubility of the carbonic acid in the blood, enabling that fluid to absorb more of the acid. Is acts similarly upon the chyme and chyle and thus becomes a prominent factor in the process of alimentation."

The same author after quoting from Baron Liebig, in reference to the function of the phosphate of sodium in preserving the equilibrium of the carbonic acid in the blood, says: "One of the most important offices of the sodic phosphate is performed in the chyle. It is a well-known fact---one repeated several times in this treatise---that while the nitrogenous digestion is an office of the stomach, carbonaceous digestion is executed in the small intestines. Physiologists were, for a long time, unable to account for all the phenomena evinced in the elaboration and absorption of the chyle. Dr. Marcet, after careful research, experiment and study, however, demonstrated that the sodic phosphate is a very efficient contributor to this process; that without it the bile and pancreatic juice could not satisfactorily complete the emulsion of the oils, fats and amylacious substances. He further proved that the oleic, stearic and margaric acids were emulsified and appropriated through its instrumentality. This discovery confirmed by the eminent physiologist, Dr. Thudicum, of London, and corroborated by the experiments of the author, clears up some of the most obscure points in the digestion of fatty matters. Fats being the nucleus around which albuminous matters are deposited, the value of phosphate of soda as a nutrient becomes apparent and the necestity of its presence in human ailment the more exalted. There are several interesting points here presented in relation to phosphoric acid viewed especially as an acid in the nutritive processes and in cell and tissue development.

The fluids which permeates the muscles and cellular tissues is acid, and this acidity is due to the presence of phosphoric acid. In opposition to this the blood is alkaline. This condition enables it to hold albumen and the albuminoids in solution. These, being carried into the minute capillaries, come in contact with the acid phosphates of potassium and ammonium are precipitated and appropriated to the different structures. In this manner the fibrin and albumen, which are liquid in the blood become solid in the tissues. If the blood be acid these would become solid in the blood, and if they be semi or non-vitalized, the deposit would be an abnormal body, and subject to tyromatous change." (From "Tuberculosis Causatious Lesions and Therapeutics," page 176.)*

It seems to me that Dr. Polk comes much nearer the true explanation of the manner tubercle is deposited and its essential character than the theory he strives so hard to maintain, that tubercle is only a dead bioplasm.

Scurvy is another disease traceable to deficiency of phosphates in the system. Wheat phosphates entirely remedies the character of salted food. The experiments with wheat phosphates on Morris Island, S. C., during the Civil War, proved that they are a specific for this disease. They were administered as a strong decoction of bran. Under their use the disease rapidly disappeared.

On theoretical ground the ordinary phosphates have been used in consumption for many years. Rayer, Cahou, Rodier, Parker and Frick found that there was almost no phosphates in the dead, from consumption. About sixty years ago Larcher, Koenig, Dupuis and Beneke held the doctrine that deficiency of the phosphates caused the disease. As the phosphates used were of the non-organismal kind nothing but failure followed their use. Recent experience with the wheat phosphates have been far more satisfactory. While the cases have been very rare in which a decided cure has been effected, the progress of the disease has, in the majority of cases, been arrested, and in those where the benefit has not been so striking the distressing symptoms have lessened and a considerable degree of comfort secured. I do not believe that any preparation of the phosphates will take the place of the hypophosphites, but that they constitute an important adjuvant to the treatment of the advanced stages in which the hypophosphites are useless---their province being in the early stage.

In the neuralgia, toothache, anæemia and disorders of pregnancy the wheat phosphates act magically, promptly relieving them. In difficult dentition, deficient ennervation and marasmus we have no remedy which compares with them in efficacy. In fact in all maladies attended with phosphatic waste they are indicated. I will give preference to any pharmaceutical manufacturer of these phosphates. I would simply suggest that the profession refuse to prescribe such as are run in the rut of secret copyrighted nostrums.—*Indiana Medical Journal.*

To prevent the loss of small screws in removing them to clean engine hand pieces and other delicate machinery, the steel screw driver may be magnetized (either by rubbing upon *one* pole of a horseshoe magnet or by passing an electric current through an insulated wire wrapped about the tool) and when removing or returning the screw it will adhere to the driver, greatly facilitating the operation.

